# Supplementary material for: Altered microRNA Transcriptome in Cultured Human Liver Cells upon Infection with Ebola Virus
Source: Int J Mol Sci. 2021 Apr 6;22(7):3792. doi: 10.3390/ijms22073792 (PMC8038836; doi:10.3390/ijms22073792)
Supplement: Supplementary file 1 [file ijms-22-03792-s001.zip › Supplementary_File/C_ GO_Analysis_Results/16-30nt_go_Mayinga-24h-Huh7_vs_Control-24h-Huh7_down.mature_mirna_targets/CC_Pvalue_tree.pdf]

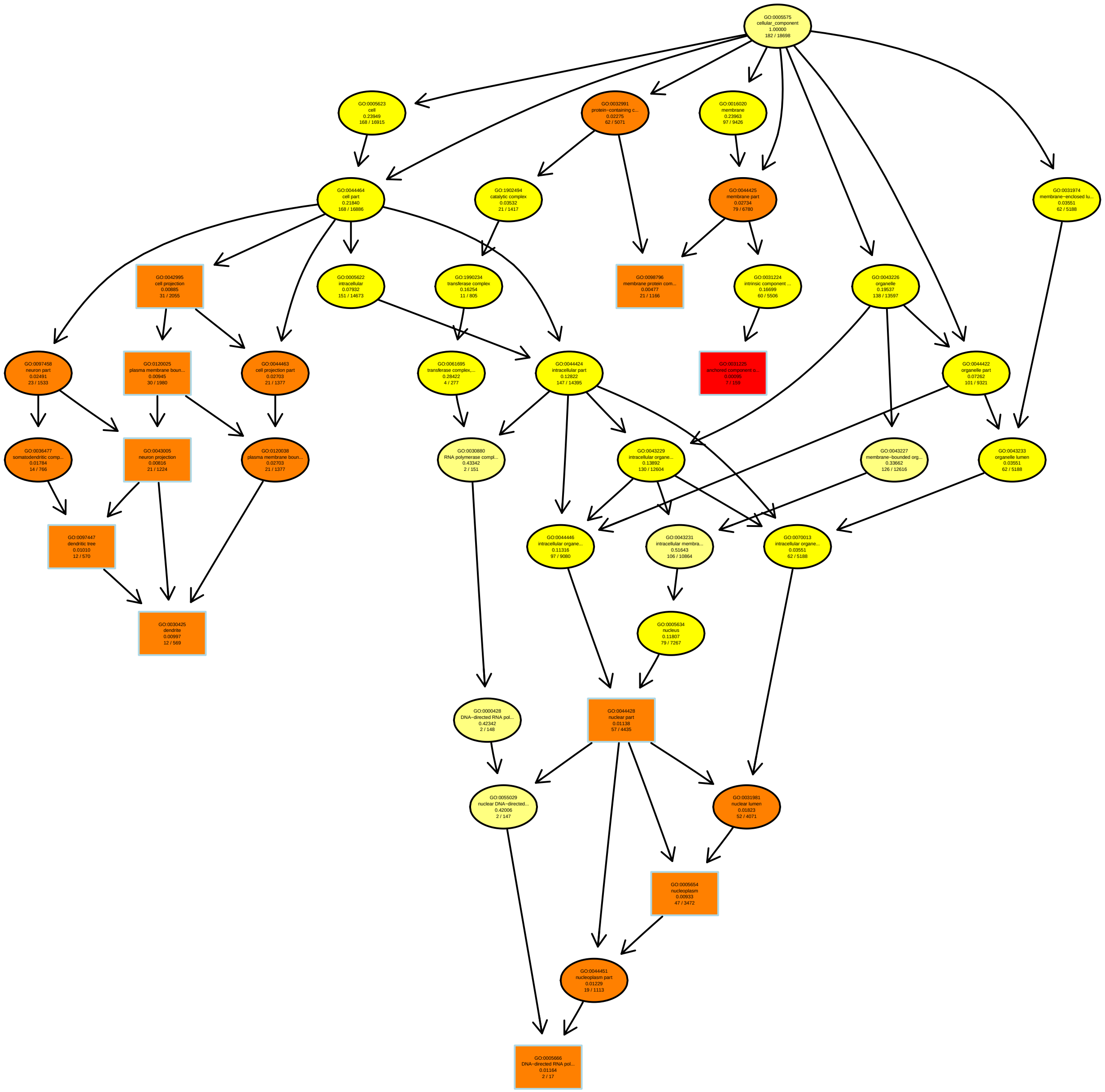

GO:0005575 cellular_component 1.00000 182 / 18698
GO:0005623 cell 0.23949 168 / 16915
GO:0032991 protein−containing c... 0.02275 62 / 5071
GO:0016020 membrane 0.23963 97 / 9426
GO:0044464 cell part 0.21840 168 / 16886
GO:1902494 catalytic complex 0.03532 21 / 1417
GO:0044425 membrane part 0.02734 79 / 6780
GO:0031974 membrane−enclosed lu... 0.03551 62 / 5188
GO:0042995 cell projection 0.00885 31 / 2055
GO:0005622 intracellular 0.07932 151 / 14673
GO:1990234 transferase complex 0.16254 11 / 805
GO:0098796 membrane protein com... 0.00477 21 / 1166
GO:0031224 intrinsic component ... 0.16699 60 / 5506
GO:0043226 organelle 0.19537 138 / 13597
GO:0097458 neuron part 0.02491 23 / 1533
GO:0120025 plasma membrane boun... 0.00945 30 / 1980
GO:0044463 cell projection part 0.02703 21 / 1377
GO:0061695 transferase complex,... 0.28422 4 / 277
GO:0044424 intracellular part 0.12822 147 / 14395
GO:0031225 anchored component o... 0.00095 7 / 159
GO:0044422 organelle part 0.07262 101 / 9321
GO:0036477 somatodendritic comp... 0.01784 14 / 766
GO:0043005 neuron projection 0.00816 21 / 1224
GO:0120038 plasma membrane boun... 0.02703 21 / 1377
GO:0030880 RNA polymerase compl... 0.43342 2 / 151
GO:0043229 intracellular organe... 0.13892 130 / 12604
GO:0043227 membrane−bounded org... 0.33662 126 / 12616
GO:0043233 organelle lumen 0.03551 62 / 5188
GO:0097447 dendritic tree 0.01010 12 / 570
GO:0044446 intracellular organe... 0.11316 97 / 9080
GO:0043231 intracellular membra... 0.51643 106 / 10864
GO:0070013 intracellular organe... 0.03551 62 / 5188
GO:0030425 dendrite 0.00997 12 / 569
GO:0005634 nucleus 0.11807 79 / 7267
GO:0000428 DNA−directed RNA pol... 0.42342 2 / 148
GO:0044428 nuclear part 0.01138 57 / 4435
GO:0055029 nuclear DNA−directed... 0.42006 2 / 147
GO:0031981 nuclear lumen 0.01823 52 / 4071
GO:0005654 nucleoplasm 0.00933 47 / 3472
GO:0044451 nucleoplasm part 0.01229 19 / 1113
GO:0005666 DNA−directed RNA pol... 0.01164 2 / 17
